# Limbus misrepresentation in parametric eye models

**DOI:** 10.1371/journal.pone.0236096

**Published:** 2020-09-24

**Authors:** Joshua Moore, Xuhan Shu, Bernardo T. Lopes, Richard Wu, Ahmed Abass

**Affiliations:** 1 Department of Mathematical Sciences, University of Liverpool, Liverpool, United Kingdom; 2 School of Engineering, University of Liverpool, Liverpool, United Kingdom; 3 College of Physical Sciences, University of Guelph, Guelph, Canada; 4 Department of Ophthalmology, Federal University of Sao Paulo, Sao Paulo, Brazil; 5 Department of Optometry, Central Taiwan University of Science and Technology, Taichung, Taiwan; 6 College of Optometry, Pacific University, Forest Grove, Oregon, United States of America; Save Sight Institute, AUSTRALIA

## Abstract

**Purpose:**

To assess the axial, radial and tangential limbus position misrepresentation when parametric models are used to represent the cornea and the sclera.

**Methods:**

This retrospective study included 135 subjects aged 22 to 65 years (36.5 mean ±9.8 STD), 71 females and 64 males. Topography measurements were taken using an Eye Surface Profiler topographer and processed by a custom-built MATLAB code. Eye surfaces were freed from edge-effect artefacts and fitted to spherical, conic and biconic models.

**Results:**

When comparing the radial position of the limbus, average errors of -0.83±0.19mm, -0.76±0.20mm and -0.69±0.20mm were observed within the right eye population for the spherical, conic and biconic models fitted up to 5mm. For the same fitting radius, the average fitting errors were -0.86±0.23mm, -0.78±0.23mm and -0.73±0.23mm for the spherical, conic and biconic models respectively within the left eye population. For the whole cornea fit, the average errors were -0.27±0.12mm and -0.28±0.13mm for the spherical models, -0.02±0.29mm and -0.05±0.27mm for the conic models, and -0.22±0.16mm and 0.24±0.17mm for the biconic models in the right and left eye populations respectively.

**Conclusions:**

Through the use of spherical, conic and biconic parametric modelling methods, the eye’s limbus is being mislocated. Additionally, it is evident that the magnitude of fitting error associated with the sclera may be propagating through the other components of the eye. This suggests that a corneal nonparametric model may be necessary to improve the representation of the limbus.

## Introduction

The tunic of the human eyeball consists of two main components, the cornea and the sclera, that meet each other at the limbus. [1] The cornea provides nearly 73% of the eye’s refractive power, [2, 3] however, the sclera is the main load-bearing structure of the eye and forms about 85% of the surface of the eye globe. [4] Although the limbus, the Latin word for ‘border’, is usually known as the corneal boundary where the cornea and the sclera are connected, it has been defined in several different ways according to the specific application. It can be defined as the junction between the cornea and the sclera from a microscopic point of view, [5] the border between the opaque sclera and the transparent cornea in terms of transparency, [6] and, when considering eye topography, it is the contour where there is a change in the curvature at the junction of eye’s two main structures. [7]

Clinically, the location of the limbus is important in the field of customisable soft contact lens fitting, as it is used when estimating the lens’s overall diameter. It is also essential in mini scleral and scleral contact lens fittings where avoidance of contact with limbal stem cells is crucial. Clinical measurement of the limbus’s location is often inaccurate and contact lens fitters tend to use the horizontal visible iris diameter (HVID) as an approximation. When used for modelling purposes, this approximation can yield significantly different geometries, thus affecting accuracy. This is primarily due to the fact that the white-to-white distance in the human eye is significantly greater in the nasal-temporal direction than in the superior-inferior direction. The human limbus diameter is not thought to vary significantly in these directions. [8] As most of the non-disposables soft contact lenses are being fitted through empirical fitting rules that depend highly on the limbus’s position, the true limbus diameter would be far more informative than the HVID. Additionally, it is also an essential parameter in the design and manufacture of single diameter disposable lens.

Many techniques have been developed for the parametric modelling of the eye. [9] The first recognised technique for modelling the corneal surface was fairly simplistic. This approach involved computing the Cartesian coordinates (x,y,z) of the corneal surface as if it were a perfect sphere with radius *R*, Eq 1. It was soon realised that spherical models were predicting higher spherical aberration and oblique astigmatism than were experimentally measured. [10, 11]

$$z=\sqrt{R^{2}-\left(x^{2}+y^{2}\right)}-R$$ Eq 1

In an attempt to eliminate the spherical aberration, a conic surface model was introduced, Eq 2. This model extended the abilities of the spherical equation by adding an asphericity coefficient *Q* to control the curvature of the corneal surface. When the asphericity *Q* is set to zero, Eq 2 is reduced to Eq 1 and the fitted corneal surface becomes spherical.

$$z=\frac{\sqrt{R^{2}-\left(x^{2}+y^{2}\right)\left(Q+1\right)}-R}{Q+1}$$ Eq 2

**Fig 1:**
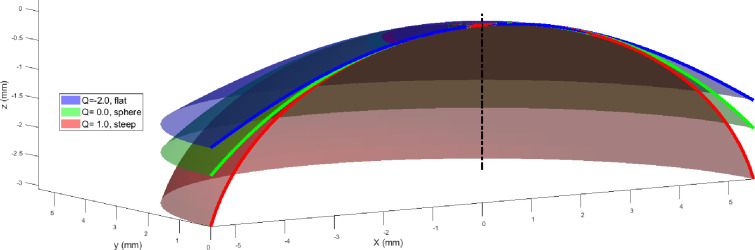
Corneal surface models generated for differing values of asphericity.

The asphericity factor *Q* is synonymous to the overall curvature of the cornea, with positive values leading to increased steepness and negative values inducing a flattening effect, Fig 1.

Both the spherical and conical models assume complete rotational symmetry and ignore the effects of astigmatism. Astigmatism occurs as a result of a phenomenon known as toricity, whereby the curvature of the nasal-temporal meridian is flatter than the superior-inferior corneal meridian. [12–14] A revision of the conic model led to the inclusion of toricity to form what is known as a biconic surface. [15] The mathematical expression that defines this model is provided in Eq 3. This model bifurcated the radius of corneal curvature into two separate values *R*_*x*_ and *R*_*y*_ representing corneal radii in the two principal directions; nasal-temporal (N-T) and superior-inferior (S-I) respectively. Two asphericity coefficients *Q*_*x*_ and *Q*_*y*_ were also included in an attempt to control the steepness of the cornea along with X-axis (N-T) and Y-axis (S-I) directions independently.

$$z=\frac{-\left(\frac{x^{2}}{R_{x}}+\frac{y^{2}}{R_{y}}\right)}{1+\sqrt{1+\left(1+Q_{x}\right)R_{x}{}^{2}x^{2}-\left(1+Q_{y}\right)R_{y}{}^{2}y^{2}}}$$ Eq 3

Additionally, a biconic eye model is often rotated around its nominal axis of rotational symmetry (Z-axis) to account for the axis of the cylindrical power of astigmatism. Rotation about the two principal directions (X and Y) can also be utilised to compensate for the difference between the visual and optical axis when measuring corneal topography. [9, 16] Due to the large number of variables included in the biconic model, its equation normally allows for a higher degree of surface control compared to the conic model (Eq 3).

Corneal conic asphericity has been reported in varied ranges down to -0.82 [17–34] (Table 1), however, biconic asphericity was reported down to -0.28 [34, 35] depending on the algorithm used and the sample size. For example, Ying provided corneal radius of 7.83mm [12] through a conic model with varying asphericity Q = -0.18:-0.3 because of astigmatism skewed distribution, [36] however, when an alternative method was introduced using corneal tangential radius of curvature instead of sagittal radius, Q was reported in a range of -0.33 to 0.12 and varied between corneal principal meridians. [37]

**Table 1:** Summary of the anterior corneal radius and asphericity as reported in the literature.

Author	Year	Number of eyes	Model	Radius (mm)	Asphericity
Mainstone [17]	1998	35	conic	R = 7.36:8.54	Q = -0.332±0.297
Holladay [18]	1999	14	conic	Not reported	Q = -0.16±0.12
Douthwaite [19]	1999	98	conic	R = 7.78:7.93	Q = 0.13:0.5
Holmes-Higgin [20]	1999	25	conic	R = 7.5:9.0	Q = -0.01:-1.44
Budak [21]	1999	287	conic	R = 6.7:9.4	Q = -0.03±0.23
Dubbelman [22]	2002	83	conic	R = 7.87±0.27	Q = -0.82±0.18
Langenbucher [40]	2002	50	biconic		
		30 keratoconic		not reported	Q_x_ = -0.42±0.21
		20 fuchs' dystrophy			Q_y_ = -0.53±0.13
Cuesta [23]	2003	92	conic	not reported	Q = -0.26±0.18
Hersh[41]	2003	11	conic	R = 6.50:6.75	Q = -0.17±0.14
Manns [24]	2004	24	conic	R = 10.15±1.39	Q = -0.64±1.85
Somani [25]	2004	278	conic	not reported	Q = -0.60:0.0
Llorente [26]	2004	46	conic		
		24 Myopic		R = 7.86±0.37	Q = -0.10±0.23
		22 Hyperopic		R = 7.97±0.30	Q = -0.20±0.17
Davis [27]	2005	643	conic		
		Myopes		R = 7.53±0.27	Q = -0.32±0.10
		Emmeropes		R = 7.54±0.24	Q = -0.35±0.10
		Hyperopes		R = 7.57±0.23	Q = -0.36±0.10
Priest [42]	2005	151	biconic	R_x_ = 7.76±0.24	Q_x_ = -0.24±0.31
				R_y_ = 7.60±0.28	Q_y_ = -0.11±0.32
Dubbelman [28]	2006	114	conic	R = 7.79±0.025	Q = -0.41±0.26
Mastropasqua [43]	2006	74	biconic	R_x_ = 6.87±0.26	Q_x_ = -0.18±0.12
				R_y_ = 6.56±0.36	Q_y_ = -0.21±0.13
Navarro [44]	2006	123	biconic	R_x_ = 7.63	Q_x_ = -0.465
				R_y_ = 7.40	Q_y_ = -0.481
González-Méijome [29]	2007	36	conic	R = 7.8	Q = -0.39±0.11
Nieto-Bona [30]	2009	118	conic		
		30 Emmetropic		R = 7.72±0.05	Q = -0.32±0.03
		56 Myopic		R = 7.40±0.03	Q = -0.36±0.02
		32 Hyperopic		R = 7.80±0.04	Q = -0.37±0.03
Piñero [31]	2010	71	conic	R = 7.89±0.31	Q = -0.29±0.09
Bottos [32]	2011	209	conic	not reported	Q = -0.27±0.12
Zhang [33]	2011	1052	conic	R = 7.80±0.25	Q = -0.30±0.12
Ortiz [45]	2012	3	biconic	R_x_ = 7.40 ± 0.07	Q_x_ = 0.10±0.01
				R_y_ = 7.53 ± 0.03	Q_y_ = -0.19±0.05
Navarro [35]	2013	407	biconic	R_x_ = 7.01:8.44	Q_x_ = -0.77:-0.10
				R_y_ = 6.85:8.31	Q_y_ = -0.81:-0.09
Bao [34]	2013	684	biconic		
		342 Right		R_x_ = 7.81±0.25	Q_x_ = -0.27±0.09
				R_y_ = 7.62±0.28	Q_y_ = -0.28±0.12
		342 Left		R_x_ = 7.81±0.26	Q_x_ = -0.27±0.08
				R_y_ = 7.62±0.27	Q_y_ = -0.27±0.12

Values of asphericity presented in Table 1 were only validated against the central corneal zone as measuring the peripheral corneal zone and the anterior sclera simultaneously has not been possible until recently. The use of an eye topographer to characterise the corneoscleral shape in vivo was not possible until the past few years as most of the topographers were not able to measure the area of the eye that covers the limbus and part of the sclera. [38] The situation has changed recently, and some newly developed topographers are able to do this by capturing the cornea and the exposed portion of the sclera, such as the Pentacam Cornea Scleral Profile (CSP) optional software and the Eye Surface Profiler (ESP). The ESP can cover up to a 20mm diameter of the eye. [39] This development in the instrumentation capabilities motivated the authors to investigate and parametrically characterise the corneoscleral zone using these recently developed topographers that can provide the anterior eye surface up to a few millimetres beyond the limbus. [8] On one hand, as most of the corneal topographers are covering up to 5mm of the corneal surface only, this study looked at the difference between fitting an eye parametric model to the 5mm central corneal radius only and fitting it to the whole corneal surface. On the other hand, as most of the eye’s parametric models were using parameters that were fitted to the central cornea, using a modern ESP surface profiler, this study is investigating how these limitations in the longstanding parametric techniques misrepresented the limbus diameter, position and how this misrepresentation can be compensated for.

## Materials and methodology

### Participants

Although no participant had been recruited specially for this study, this record review study was conducted according to the tenets of the Declaration of Helsinki and was approved by the IRB (Institutional Review Board) and Human Ethics Committee of the Federal University of São Paulo (UNIFESP, SP, Brazil). The study utilised a collection of secondary data where healthy eyes were selected to be processed. According to the University of Liverpool’s Policy on Research Ethics, ethical approval was unnecessary for secondary analysis of fully anonymised data. The study included 125 subjects aged 22 to 65 years (36.5 mean ±9.8 STD), 66 females and 59 males. Participants suffering from ocular diseases or having a history of trauma or ocular surgery were excluded. The data were collected and anonymised at Brighten Optix Corporation in Taipei, Taiwan where participants (most of them soft contact lenses wearers) were told not to wear soft contact lens for two weeks before the topography measurement, and those who were wearing rigid gas-permeable (RGP) contact lens were asked not to wear them for four weeks before the scan.

The ESP measurement technique involves using Moire fringes reflected from the surface of the tear film. Fixation was established by asking the subject to observe the red-cross target on the faceplate of the instrument while this was viewed by the clinician on the computer monitor. The alignment was achieved by identifying the centre point of two corneal images of lights originating from the instrument. The red-cross was then aligned with this central point and a reading initiated. Once this had been done, the subject was directed to sit back and one unpreserved lubricating drop (Lubrisitil, 1mg/mL sodium hyaluronate) was instilled into the lower fornix. This was followed by the application of fluorescein in the upper and lower fornix to maximise coverage. The subject was directed to blink a couple of times, and the level of coverage was then checked visually before proceeding further. The subject was instructed to open their eyelids as wide as possible while a measurement was being taken. This was to ensure good data coverage beyond the limbal zone. The measurement was then repeated twice and the best scan, in terms of coverage range and quality as assessed by the ESP software, was used. Participants’ eye shape clinical parameters are reported in Table 2 as measured by the ESP built-in interface (see S1 Data for full details).

**Table 2:** Participants’ eye shape clinical parameters as measured by the ESP.

	Corneal shape parameters (M±STD)				Simulated keratometry (Sim-K) parameters (M±STD)			
	HVID (mm)	Astigmatism (Dioptre)	Axis (Degree)	Sphere (Dioptre)	Astigmatism (Dioptre)	Axis (Degree)	Flat Radius (mm)	Steep Radius (mm)
Right eyes (OD)	11.9±0.4	-1.6±0.7	99.5±43.2	43.1±1.7	-2.5±1.1	100.4±43.5	8.3±0.4	7.9±0.3
Left eyes (OS)	11.9±0.4	-1.9±0.8	100.8±42.5	43.1±1.7	-2.9±1.2	95.9±44.1	8.4±0.4	7.9±0.4

### Data collection and processing

**Fig 2:**
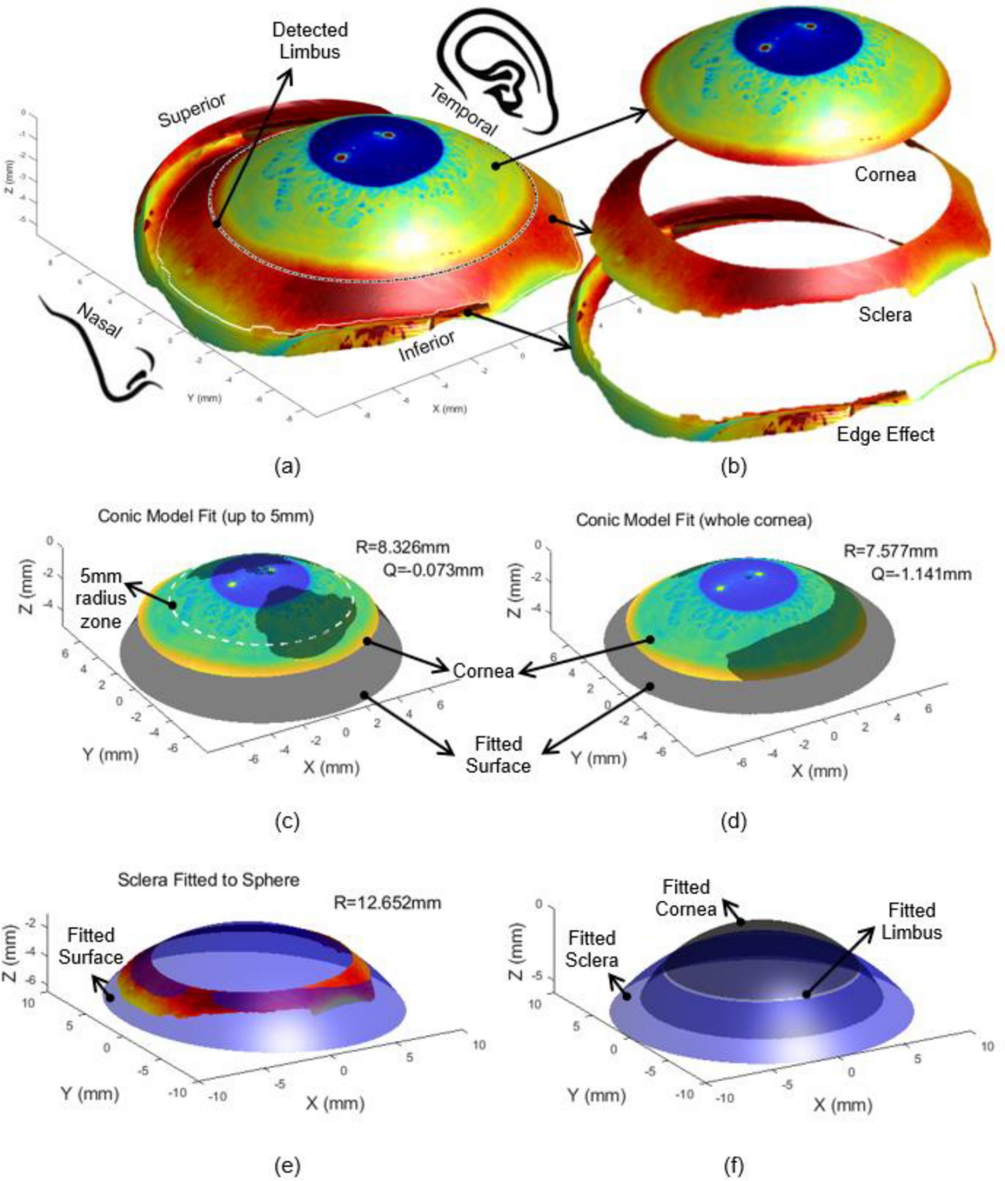
Left eye of a 25 year old male subject where the eye’s front image was projected on the surface for displaying purpose (a) as scanned by the ESP, where the limbus was detected in three-dimensions [8] (b) after being processed to split cornea, sclera and the edge effect artefacts following Abass’s method. Two edge detection strategies were used simultaneously to cut the edge of the eye’s surface data at the border between the authentic eye surface and the artificial boundaries which result from interference of tears, eyelid edges or lashes [46] (c) corneal conic fit up to 5mm, (d) whole corneal conic fit, (e) sclera fitted to a sphere, (f) Fitted limbus as constructed by the intersection of the fitted surfaces representing the cornea and the sclera.

The data was exported from the ESP software in MATLAB (MathWorks, Natick, USA) binary data container format (*.mat). The eye surface data was processed by custom-built MATLAB codes entirely independent from the built-in ESP software digital signal processing (DSP) algorithms. Firstly, each eye surface was freed of edge-effect artefacts following Abass’s three-dimensional non-parametric method [46], Fig 2A and 2B. Three-dimensional surfaces were fitted to the ESP topographical data using the spherical, conic and biconic techniques outlined in the introduction. In each case, the sclera was modelled as a sphere with its radius calculated as the best fit radius by the least-squares method. [47] The spherical corneal models were generated by deducing the radius and the centre that was necessary to fit the ESP data to a perfect sphere, and then using this value to calculate the modelled surface.

The fitting of the conic and biconic surfaces introduced an added complexity. In order to gain values of the required variables, the Levenberg-Marquardt nonlinear least-squares algorithm (LMA) was utilised. [48] LMA is a robust technique for solving nonlinear curve fitting problems by searching for a solution that minimises the fitting error. [49] The algorithm involves defining an objective function based on either the conic or biconic models and then deducing the values of asphericity and corneal radius that minimise the root mean square (RMS) error between the eye surface and the fitted surfaces, [50] Fig 2C and 2D. As the algorithm requires an initial guess of the parameters, the initial estimates of the corneal radius of curvature and asphericity used in the conic fitting process were 7.8mm and 0.0 respectively. [51] The biconic model required initial values of radii of curvature in the two principal directions R_x_, R_y_ and the angle of rotation about the Z-axis (*θ*_*z*_) that is required to account for astigmatism and the asphericity in the two principal directions. The two radii were given initial guess values of 7.8mm and all other variables were initially set to zero.

Following the calculation of the aforementioned variables, the surfaces were generated using the spherical, conic and biconic surface definitions. An additional rotation about the Z-axis was included for the biconic surfaces and achieved by multiplying the Cartesian coordinates, for each point on the surface, by the matrix for rotation about the Z-axis [52]. For a rotation angle of *θ*_*z*_, this matrix is given by:

$$W_{z}\left(\theta_{z}\right)=\left[\begin{array}{ccc} \cos\theta_{z} & -\sin\theta_{z} & 0\\ \sin\theta_{z} & \cos\theta_{z} & 0\\ 0 & 0 & 1 \end{array}\right]$$ Eq 4

Following the elemental rotation rule, the rotated coordinates of the corneal surface *X*_*r*_, *Y*_*r*_ and *Z*_*r*_ were calculated as

$$\left[\begin{array}{lllll} x_{r1} & x_{r2} & x_{r3} & \cdots & x_{rn}\\ y_{r1} & y_{r2} & y_{r3} & \ldots & y_{rn}\\ z_{r1} & z_{r2} & z_{r3} & \ldots & z_{rn} \end{array}\right]=W_{z}\left(\theta_{z}\right)*\left[\begin{array}{ccccc} x_{1} & x_{2} & x_{3} & \ldots & x_{n}\\ y_{1} & y_{2} & y_{3} & \ldots & y_{n}\\ z_{1} & z_{2} & z_{3} & \ldots & z_{n} \end{array}\right]$$ Eq 5

**Fig 3:**
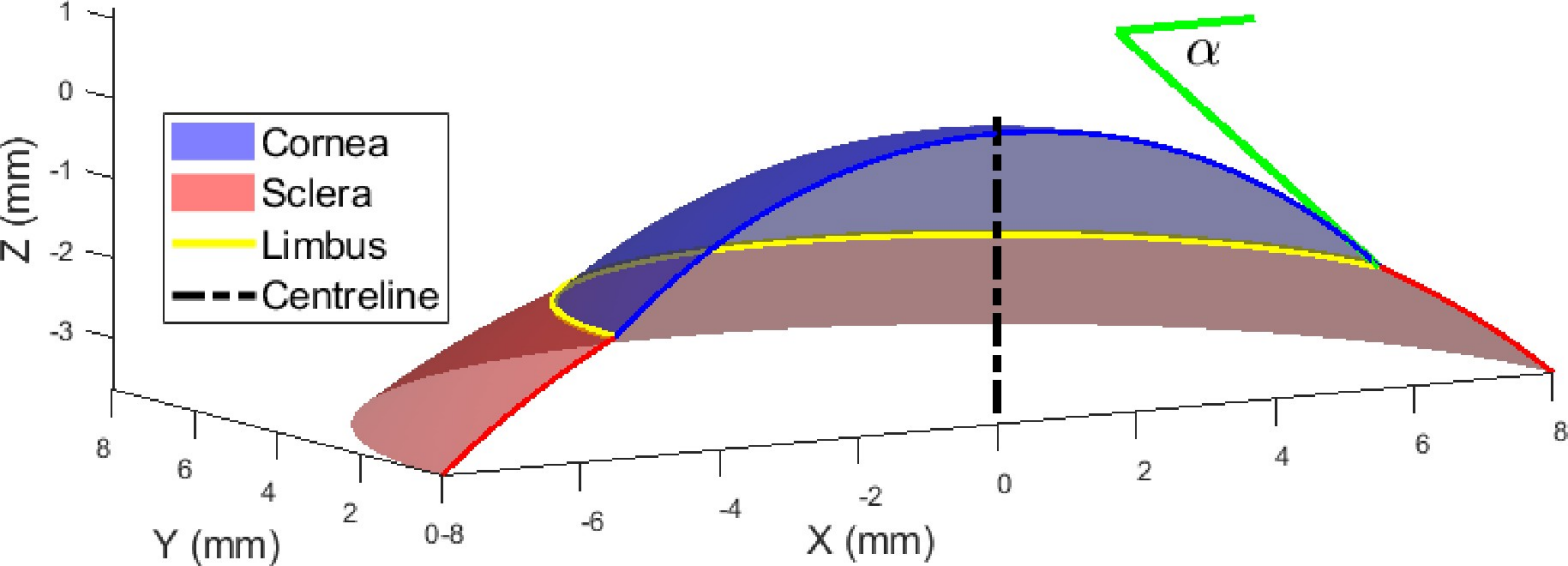
Graphical representation of the angle that the corneal surface makes at the point of connection to the limbus. The tangent angle at the limbus is represented by α.

Before moving to the next processing stage, the origin position of each levelled eye’s surface was shifted to the highest point of the limbus-levelled eye surface (apex). The process of fitting the sclera to the spherical surface was accompanied by the calculation of the scleral radius and centre offset, relative to the corneal apex, Fig 2E. Each of the surfaces was plotted and compared directly to the surface outputted by the ESP through different geometrical observations. These observations were the radius of curvature, the positioning of both the detected limbus and the fitted one, Fig 2A and 2F, and the angle that the corneal surface makes at the limbus (α), Fig 3. The radii of curvature values were fairly simple to compute through the coordinates that define the surface, however, deducing the geometrical properties of the limbus required an alternative approach. To compute the limbal tangent angle α, Fig 3, the tangent of each measured eye surface was computed, for each meridian through the first derivative of the surface’s z-values in the polar coordinate system (z_p_), with respect to the instantaneous radius (r) at the same meridian around 360° (Eq 6). The tangent angle at the limbus position was then determined by applying a piecewise Cubic Hermite interpolating polynomial at the limbal position of each meridian.

$$\alpha_{t}=\tan^{-1}\frac{dz_{p}}{dr}$$ Eq 6

The position of the detected limbus on the corneal surface was identified through the use of a three-dimensional non-parametric method for limbus detection introduced by Abass et al. in 2018 [8]. Briefly, the algorithm is based on the fact that the cornea and the sclera have different curvatures [53] and the limbus is the boundary where the corneal curvature changes to match that of the sclera. [54] Therefore, the position of the limbus was detected by locating the turning point of the raw elevation 2^nd^ derivative at each meridian. This limbal position is referred to as the detected limbus position in this manuscript. The intersection of the fitted corneal and scleral surfaces did not necessarily occur in the same radial or axial position as the positions measured experimentally. The difference between the fitted and actual limbal positions was calculated and presented in this study.

For each of the geometrical properties, the associated fitting errors were calculated by taking the actual value and subtracting it from the value present in the fitted model. Using this approach allowed for the understanding of whether the fitting technique was over or underestimating the geometry.

### Example of finite element modelling

In order to demonstrate the effect of misrepresenting the limbus area in finite element modelling, the left eye of a 25 year old male subject, shown in Fig 2, was modelled using two different techniques. The first model was built by using the geometry of the eye as measured and extracted from the ESP, however the second was constructed using the parametric values obtained by fitting the central 5mm radius of the cornea to a conic model (R = 8.3258mm, Q = -0.0729). In both cases, the sclera was fitted by minimising the RMS error in the axial direction to 12.652mm sphere after separating the scleral portion of the topography data, Fig 2B. Central corneal thickness (CCT) was taken as 0.55mm [55] which increased to 0.70mm and 0.56mm at the peripheral corneal zone and scleral equatorial ring respectively. [56, 57] Additionally, at the eye posterior pole the thickness was taken as 0.84mm. [58] Eight-node first-order continuum solid hybrid brick elements ‘C3D8H’ were used in two layers of elements to build the eye model with an aspect ratio close to one in ABAQUS (Dassault Systèmes, Vélizy-Villacoublay, France) finite element software package licenced to the University of Liverpool, UK. The in-vivo human eye globe topography is measured whilst the eye is stressed due to the intraocular pressure (IOP) hence, cannot be used for modelling without pre-processing. [59] To achieve the stress-free geometry that is needed for modelling purposes, eye globe models were initially built with the inflated dimensions, then the stress-free version of each model was calculated separately by following the iteration-based method presented in [60]. In each case the stress-free model was computed by considering an IOP of 15mmHg and a maximal node position error less than 10^-4^mm. Once the stress-free models were determined, they were loaded up to IOP = 15mmHg while the equatorial nodes were bounded in the axial direction and the Von Mises [61] stress distribution was monitored through ABAQUS colour-scale. In order to keep the focus on the geometrical effect only, single 1^st^ order (N = 1) Ogden hyperelastic material model was used for the whole eye globe where the material parameters were set to average values of μ_1_ = 0.07 MPa and α_1_ = 110.836 based on our previous knowledge. [62–64] The Ogden constitutive strain energy equation can be expressed as [65]

$$U=\overset{N}{\underset{i=1}{\sum }}\frac{2\mu_{i}}{\alpha_{i}^{2}}\left({\bar{\lambda }}_{1}^{\alpha_{i}}+{\bar{\lambda }}_{2}^{\alpha_{i}}+{\bar{\lambda }}_{3}^{\alpha_{i}}-3\right)$$ Eq 7

Where *U* is the strain energy; *μ*_*i*_ and *α*_*i*_, are material parameters; ${\bar{\lambda }}_{i}$ are the deviatoric principal stretches in principal directions (i = 1,2 and 3). Accordingly, the Von Mises stress *σ*_*v*_ can be expressed as [66]

$$\sigma_{v}=\sqrt{\frac{{\left(\sigma_{1}-\sigma_{2}\right)}^{2}+{\left(\sigma_{2}-\sigma_{3}\right)}^{2}+{\left(\sigma_{3}-\sigma_{1}\right)}^{2}}{2}}$$ Eq 8

where *σ*_*j*_ are the principal stresses (j = 1,2 and 3).

### Statistical analysis

The results were subjected to statistical analysis through the use of the MATLAB Statistics and Machine Learning Toolbox. A significance level of 5% was set and the probability of the null hypothesis (p-value) was computed using a paired-sample t-test. [67] This calculation was carried out on pairs of data sets to ensure that the observed effects were not occurring as a result of sampling error. Due to the choice of significance level, the observed effects were deemed significant if they achieved a p-value lower than 0.05. Additionally, root mean square fitting errors, RMS, were computed using the z coordinates of the clinical and modelled data as

$$RMS=\sqrt{\frac{1}{N}\overset{N}{\underset{i=1}{\sum }}{\left(Z_{i_{clinical}}-Z_{i_{model}}\right)}^{2}}$$ Eq 9

where N is the number of points used in the RMS calculation.

## Results

### Corneal radius of curvature

**Fig 4:**
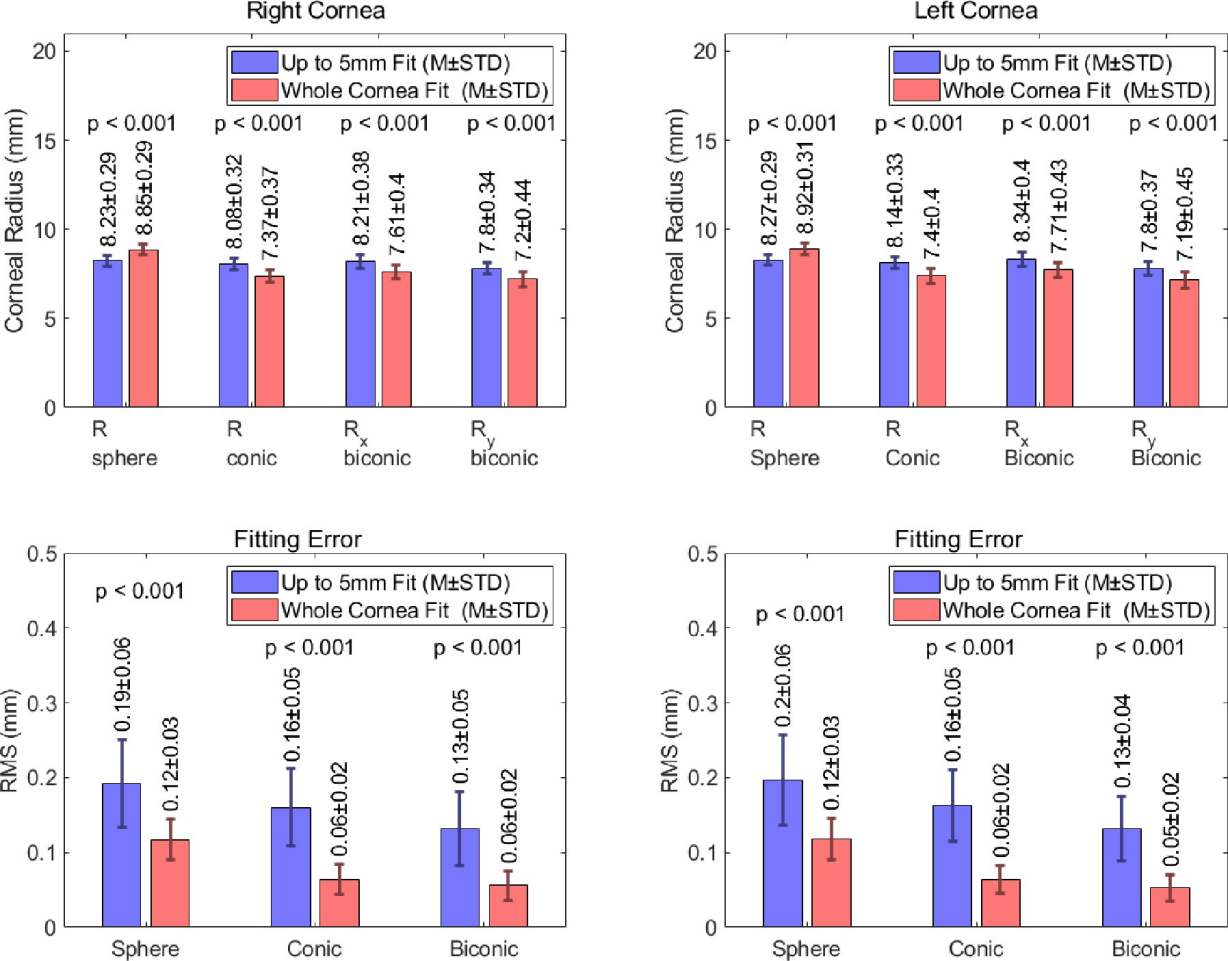
Corneal radius of curvature values measured from corneal models produced using spherical, conic and biconic fitting procedures. Fitting errors representing the RMS error between the values produced by the model and those obtained clinically.

The radii of curvature measured from spherical, conical and biconical fitted corneas are shown in Fig 4. A representation of the accuracy of these fitting techniques is also presented as the RMS error between the radii produced by fitting and the corresponding truly calculated values. For an up to 5mm fit, the spherical corneas produced RMS errors of 0.19±0.06mm and 0.2±0.06mm in the right and left eye populations respectively. As would be expected, this error is reduced considerably when the geometry is modelled using measurements from the whole cornea. This approach yields an RMS error 0.12±0.03mm in both the right and left population, for the spherical corneas.

The addition of the asphericity factor in the conic fitting procedure leads to a further reduction in the associated error. When this approach is considered, RMS readings of 0.16±0.05mm and 0.06±0.02mm were observed, for both the right and left eye populations, using an up to 5mm fit and whole cornea fit respectively. The biconic approach, with an up to 5mm fit, yielded RMS fitting errors 0.13±0.05mm and 0.13±0.04mm in the right and left eye populations respectively. When the biconic models were fitted to the whole cornea, the RMS fitting error fell to 0.06±0.02mm and 0.05±0.02mm in the right and left eye samples respectively.

### Scleral fitting

**Fig 5:**
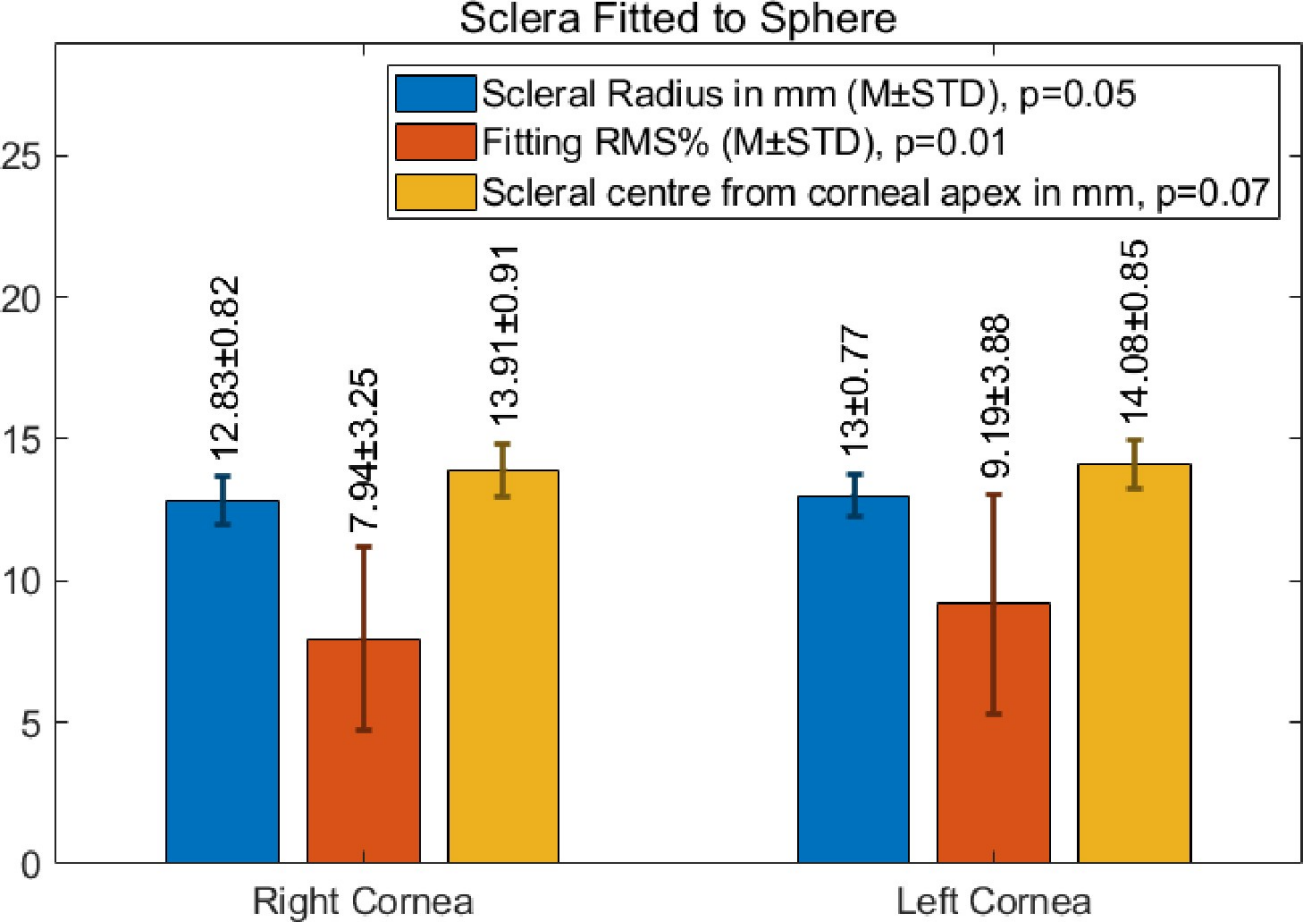
Mean scleral radius, distance from the scleral centre to the corneal apex and fitting error (RMS) for fitting the sclera to a sphere.

The mean values of scleral radius and central offset and the overall RMS scleral fitting error are presented in Fig 5. The average scleral radius for the fitted surface was 12.83±0.82mm and 13.00±0.85mm in the right and left eye populations respectively. As would be expected, the offset of the sclera’s centre with respect to the corneal apex was greater than the radius. The magnitude of the offset was 13.91±0.91mm in the right eye population and 14.08±0.85mm in the left. The error associated with the scleral fitting was statistically consistent across both eye (p = 0.01) and took values of 0.0794±0.0325 and 0.0919±0.0388 in the right and left eye populations respectively.

### Limbal positioning

**Fig 6:**
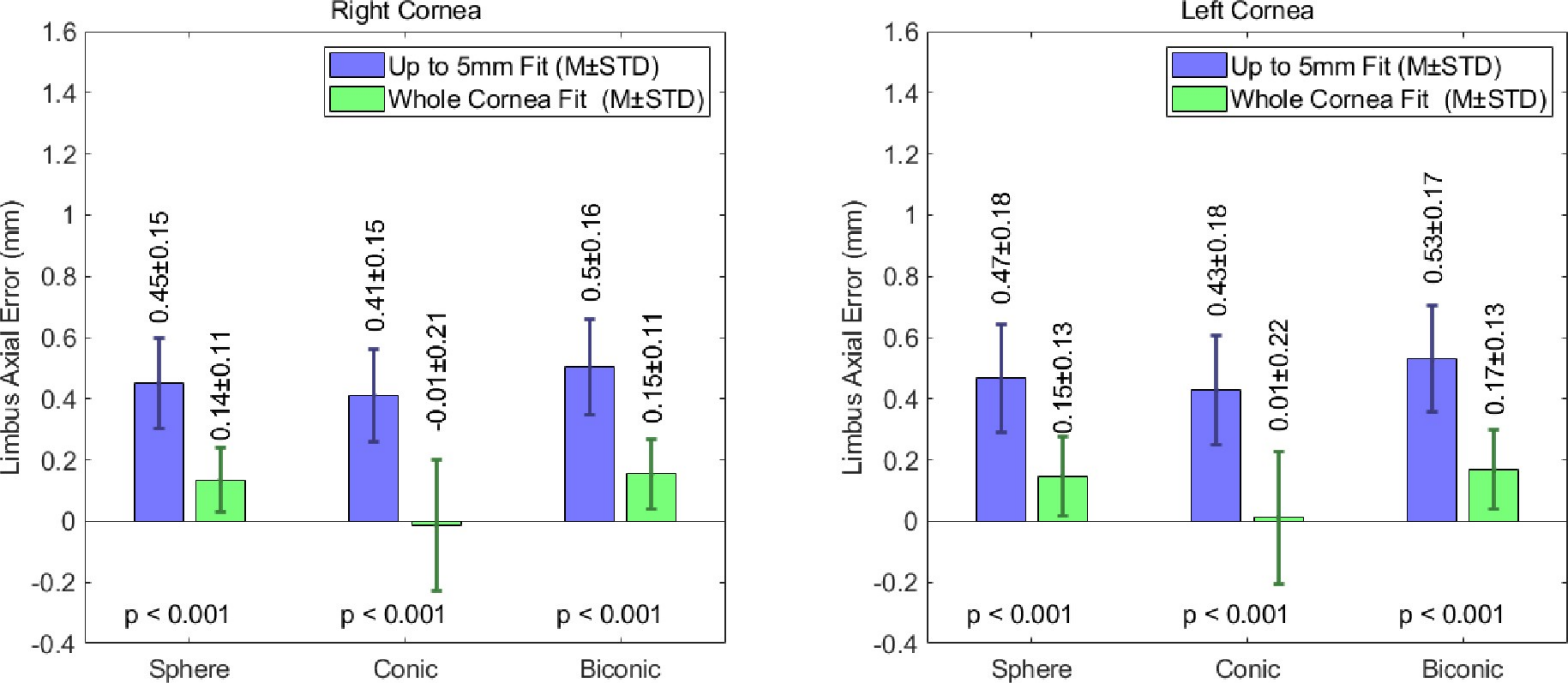
Limbus axial position error of fitted limbus compared to detected limbus when spherical, conic and biconic fitting modelling techniques.

The fitting errors for the limbus’s axial position, introduced through the spherical, conic and biconic fitting models, are presented in Fig 6. This data demonstrates that the limbus’s axial position is being consistently overestimated. A spherical fit, of up to 5mm, introduced axial position errors of 0.45±0.15mm and 0.47±0.18mm in the right and left cornea populations respectively. The adoption of a conical approach, with the same fitting radius, yielded limbal axial position errors of 0.41±0.15mm for the right eye samples and 0.43±0.18mm for the left. The increase in complexity associated with the biconic fitting procedure did not lead to an increase in accuracy when considering the positioning of the limbus. For an up to 5mm fitting radius, the RMS limbus axial position error was 0.50±0.16mm and 0.53±0.17mm for the right and left eyes respectively.

The extension of the fitting radius to the whole cornea led to a general decrease in the fitting error. When fitted to the entire cornea, the spherical models produced axial position errors of 0.14±0.11mm and 0.15±0.13mm in the right and left eyes respectively. The RMS fitting error was almost eliminated when the conic models were extended to a whole cornea fit. In right and left eye populations, the RMS limbus axial position errors were -0.01±0.21mm and 0.01±0.22mm respectively. As was observed for the 5mm fit, the whole cornea biconic models were the least accurate when considering the elevation of the limbus. The RMS limbus axial position errors for these models were 0.15±0.11mm and 0.17±0.13mm for the right and left corneas respectively.

**Fig 7:**
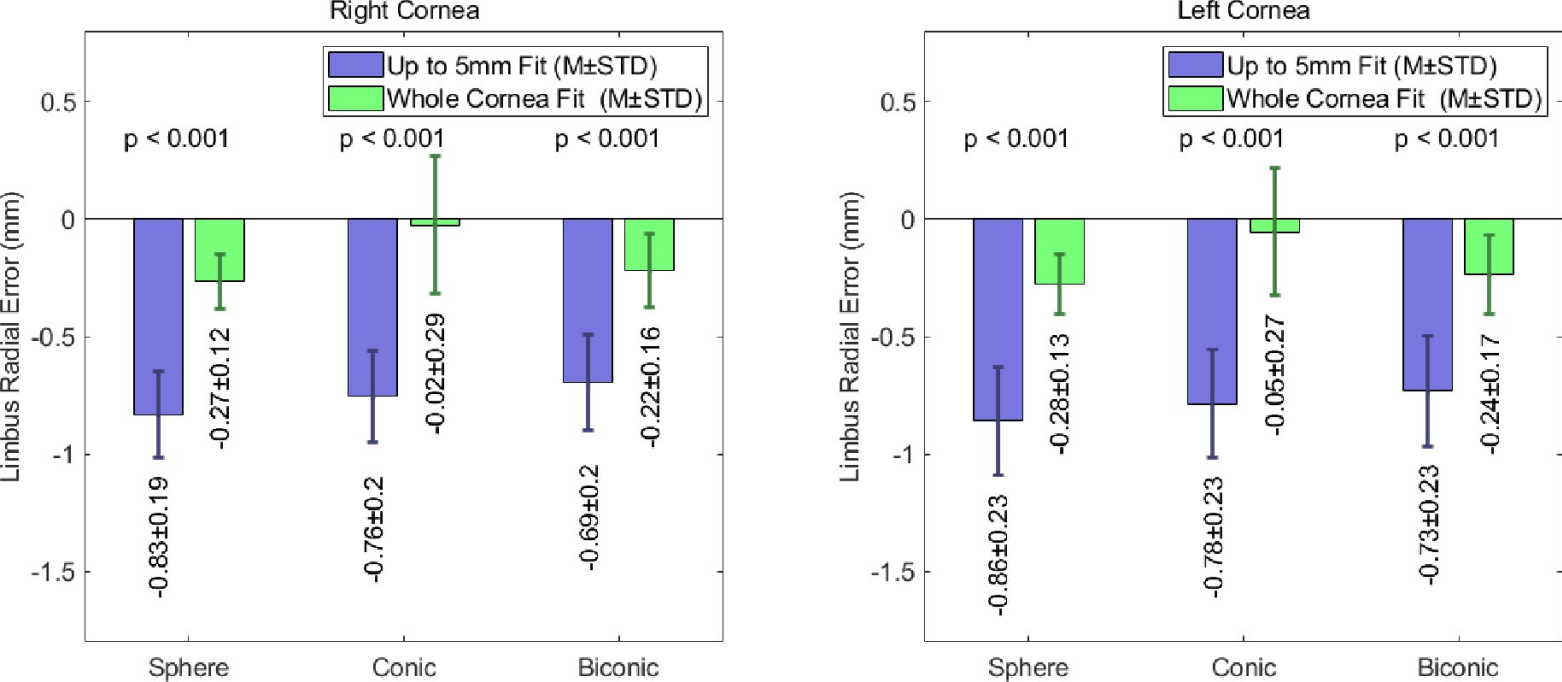
Limbus radial position error of fitted limbus compared to detected limbus when spherical, conic and biconic fitting modelling techniques limbus, for spherical, conic and biconic modelling techniques.

The accuracy of the limbal positioning was also considered by comparing the radial positions of the limbus in the numerically generated models to the experimentally measured data. The fitting errors associated with this comparison are represented graphically in Fig 7, and indicate that the radial positioning of the limbus in the fitted model is consistently smaller than the actual readings. For an up to 5mm fit, the models in which the cornea was modelled as a sphere produced RMS limbal radial position errors of -0.83±0.19mm and -0.86±0.23mm for the right and left eye populations respectively. For the same fitting radius, this error was reduced when a conic modelling technique was adopted. This approach yielded RMS limbus radial position errors of -0.76±0.20mm in the right eye population and -0.78±0.23mm in the left. The radial position error was reduced further when a biconic approach was considered. For an up to 5mm fit, the spheroid biconic corneas produced RMS errors of -0.69±0.2mm and -0.73±0.23mm for right and left corneas respectively.

As noted previously, the use of whole a cornea fit vastly improves accuracy when considering the positioning of the limbus. When adopting this radius of fit, the spherical and biconic corneas produced similar magnitudes of limbus radial position error. The limbus radial position errors for the spherical and biconic corneas were -0.27±0.12mm and -0.22±0.16mm for the right corneas, and -0.28±0.13mm and -0.24±0.17mm respectively in the left cornea population. These errors are considerably higher than in the conic models, where the radial fitting error values were -0.02±0.29mm and -0.05±0.27mm in the right and left corneas respectively.

### Limbal angle

**Fig 8:**
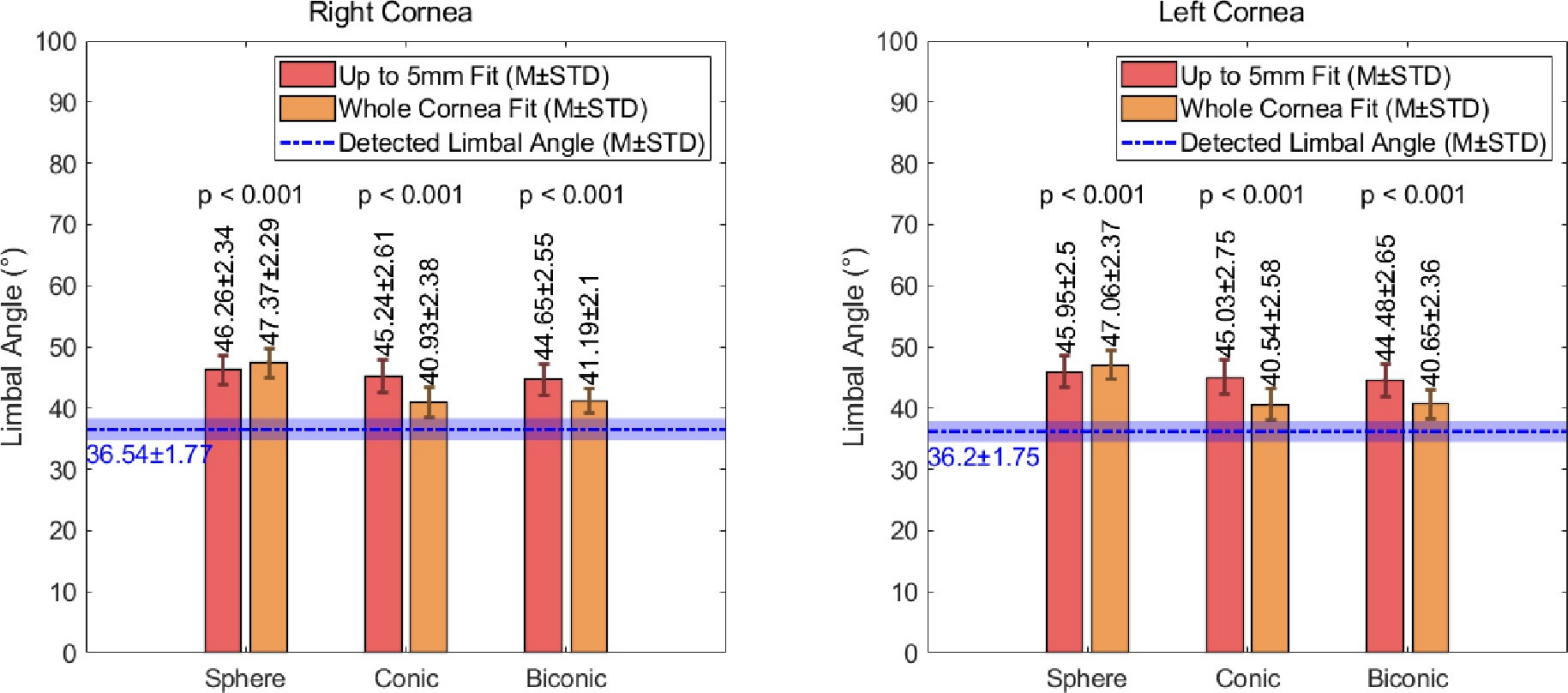
Observed values of the angle at the limbus for corneal surfaces fitted using spherical, conic and biconic techniques.

The results generated by measuring the angle at the limbal-corneal connection are presented in Fig 8. In the data generated using a fit with a radius of up to 5mm, the right corneas yielded mean angles of 46.26±2.34°, 45.24±2.61° and 44.65±2.55° for the spherical, conic and biconic fits respectively. The mean angles observed in the left corneas were 45.95±2.50°, 45.03±2.75°, 44.48±2.65° for the spherical, conical and biconical fitting techniques respectively. Despite the general consistency in these values, they each differ significantly to the experimentally measured values of 36.54±1.77° in the population of right eyes and 36.20±1.75° in the left eye population. When a whole cornea fitting approach is utilised, an improvement is observed in the conical data, with average limbal angles of 40.93±2.38° and 40.54±2.58° for the right and left eyes respectively, and the biconical data, with average right and left limbal angles of 41.19±2.10° and 40.65±2.36° respectively. Despite this, improvements were not achieved when the spherical fitting approach was utilised for the whole cornea. In this case, the average limbal angle increased to 47.37±2.29° in the right eyes and 47.06±2.37° in the left eyes, when compared to the up to 5mm fit (see S2 Data for full details).

### Stress concentration effect

**Fig 9:**
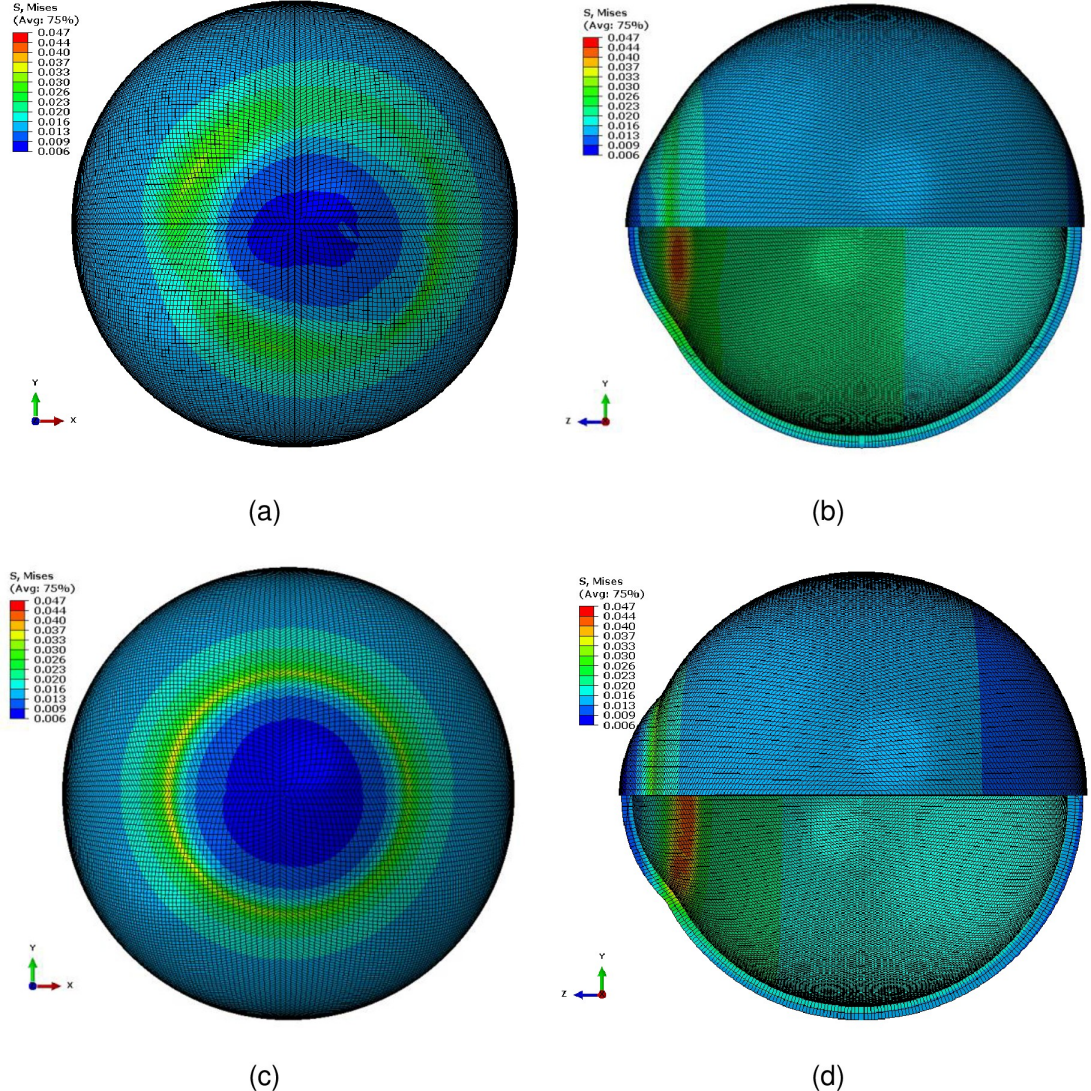
Von Mises stress distribution in MPa of two finite element models for the same subject plotted in Fig 2, (a,b) with the corneal geometry as measured by the ESP, (c,d) with corneal geometry constructed using the paramedic values obtained by fitting the central 5mm radius of the cornea to a conic model with R = 8.3258mm and Q = -0.0729.

The distribution of the Von Mises stress for the example investigated in this study showed a smooth stress distribution when the eye globe was modelled with the actual anterior geometry as measured by the ESP, Fig 9A and 9B. Despite this, the Von Mises stress distribution showed a clear stress concentration ring around the limbus when the anterior geometry constructed using paramedic values obtained by fitting the central 5mm radius of the cornea to a conic model with R = 8.3258mm and Q = -0.0729, Fig 9C and 9D. As both models were built using a single set of material parameters, it could be deduced that the geometry of models was causing this stress concentration (Abaqus odb files for the clinical limbus representation as measured by the ESP and the conic model representation are available as S3 Data and S4 Data respectively).

## Discussion

In this study, the geometrical errors induced by spherical, conic and biconic fitting techniques are quantified for a population of 270 healthy corneas. This paper focuses specifically on the effect of using these techniques on the representation of the limbus. Results were achieved by measuring the eye topography using the ESP device, fitting the surface to the aforementioned parametric models and computing the geometrical differences between the models and the surfaces provided by the ESP. As the ESP is capable of measuring corneal topography at varying distances from the centre of the cornea, the effect of fitting the models to surfaces with radii of up to 5mm and those fitted to the whole cornea were also considered.

The errors associated with the corneal radius of curvature demonstrate that, in all cases, if the whole corneal surface is used for fitting purposes, the accuracy of the results is improved significantly (p<0.001 in all cases). It is also evident from the up to 5mm fit data, that a biconic approach should be utilised to minimise the associated fitting error.

Inter-eye differences were observed in a few ocular parameters. For instance, ocular tilt during the fixation process tends to be higher in the right eye. [16] Regarding the axial length, Mahroo, et al have observed in a large twin cohort that right eyes were slightly but significantly longer than left eyes; and that this difference was reverted for left-eye dominants, the minority of that cohort. [68] In the present study, a slightly but significantly lower RMS fitting error was observed in right eyes. Further investigations are necessary to better understand this apparent more regularity in the right side, such as considering the eye dominance and tilt.

Unlike Tan et al [69] who suggested a sum of the squared orthogonalized residuals (SSRo) nondimensional metric for quantifying the limbus, this study presents dimensional assess for the axial, radial and tangential limbus position misrepresentation when parametric models are used to represent the anterior eye. The presented evaluation is modelling-friendly as it describes physical numerical values and their units of measurement.

The limbus is an area that incurs highly concentrated stresses when conducting finite element analysis. [50] A possible source of this concentration is the abrupt change in curvature at the limbus that is directly related to the limbal angle. When modelling the limbus, this angle is often too large and therefore does not represent reality. These issues indicate that an accurate representation of limbus geometry is vital for both the stability and accuracy of ocular models.

When performing the finite element analysis, one of the major issues was the poor quality of the modelled geometry, which led to instabilities in the analysis. The sharp edges that are introduced when considering a false representation of the limbus are a source of numerical singularity. At these locations the analysis algorithm can fail to predict a sensible result, even with a fine mesh. Whilst finite element modellers tend to resolve these artificial stress concentrations by replacing sharp edges with fillets and chamfers this leads to a significant misrepresentation when considering the ocular globe. Many of the ocular models that are based on the parametric representation of the eye were exposed to this side effect. [70–73]

The values of the limbal tangent angle, calculated for the different modelling techniques, reinforce the suggestion that increasing the area of the cornea used for fitting purposes provides a reduction in the induced fitting error (p<0.001, Table 3). It is also evident that, for both fitting sizes, the accuracy of the conic approach matches that of the biconic (p<0.05 in all cases). This suggests that the biconic technique may not always be necessary to achieve the required accuracy. The data also indicates that if the most accurate approach is utilised, whereby a whole corneal surface is fitted using a conic or biconic technique, a consistent correction factor of -4° is sufficient to provide a significantly improved angle for modelling purposes. For all other approaches, a correction factor of -9° will provide an improved representation of the limbus.

**Table 3:** Values of p-value calculated through significance testing, a p-value < 0.05 indicates significance.

							Right Cornea
							Left Cornea
**Corneal Radius of Curvature RMS**							
Up to 5mm Fit				Whole Cornea Fit			
	Sphere	Conic	Biconic		Sphere	Conic	Biconic
Sphere		p < 0.001	p < 0.001	Sphere		p < 0.001	p < 0.001
Conic	p < 0.001		p < 0.001	Conic	p < 0.001		p < 0.001
Biconic	p < 0.001	p < 0.001		Biconic	p < 0.001	p < 0.001	
**Limbal Tangent Angle (α)**									
Up to 5mm Fit					Whole Cornea Fit				
	Actual	Sphere	Conic	Biconic		Actual	Sphere	Conic	Biconic
Actual		p < 0.001	p < 0.001	p < 0.001	Actual		p < 0.001	p < 0.001	p < 0.001
Sphere	p < 0.001		p < 0.001	p < 0.001	Sphere	p < 0.001		p < 0.001	p < 0.001
Conic	p < 0.001	p < 0.001		p < 0.001	Conic	p < 0.001	p < 0.001		p = 0.006
Biconic	p < 0.001	p < 0.001	p < 0.001		Biconic	p < 0.001	p < 0.001	p = 0.004	
**Limbal Axial Position Error**							
Up to 5mm Fit				Whole Cornea Fit			
	Sphere	Conic	Biconic		Sphere	Conic	Biconic
Sphere		p < 0.001	p < 0.001	Sphere		p < 0.001	p = 0.002
Conic	p < 0.001		p < 0.001	Conic	p < 0.001		p < 0.001
Biconic	p < 0.001	p < 0.001		Biconic	p = 0.005	p < 0.001	
**Limbal Radial Position Error**							
Up to 5mm Fit				Whole Cornea Fit			
	Sphere	Conic	Biconic		Sphere	Conic	Biconic
Sphere		p < 0.001	p < 0.001	Sphere		p < 0.001	p < 0.001
Conic	p < 0.001		p < 0.001	Conic	p < 0.001		p < 0.001
Biconic	p < 0.001	p < 0.001		Biconic	p < 0.001	p < 0.001	

The positioning of the limbus and its associated fitting error have also been presented in this paper by considering both its axial and radial position. The data representing the elevation of the limbus (axial position) demonstrate strikingly different behaviour to the previously considered geometric properties. This is represented by the consistently higher fitting error produced by the biconic models when compared to the other fitting techniques. This discontinuity is not amended by increasing the area of the cornea that is used for fitting. This suggests that the use of a biconic modelling technique may hinder the modelling of the limbus greatly. In the measurements gained from the two fitting areas, the conic approach provided the most accurate values of limbal elevation and, if used solely in conjunction with a whole cornea fit, the conic fitting technique can reduce the fitting error to magnitudes that can be considered negligible. For both fitting radii, it was generally observed that the fitted models were overestimating the overall limbal elevation. A correction factor is not necessary if a conic model is utilised with the higher fitting radius, however if the model is spherical or biconic, a constant correction factor of -0.15mm can be applied if used in conjunction with the whole cornea fit. If a smaller fitting radius is used, a correction factor of -0.45mm is suitable for each of the modelling techniques.

The fitting errors that correspond to the limbus’s radial position indicate that, in all cases, the radial position of the limbus in the fitted models was lower than the actual measurements. For the up to 5mm fit, the spherical and biconic techniques produce the largest and smallest fitting errors respectively. This relation is not observed when the models are fitted to the whole cornea and, as was present in the limbal elevation data, the conic technique produces fitting errors that are considerably lower than those produced using spherical or biconical methods (p<0.001 in both cases). A correction factor of +0.75mm will lead to significantly lower fitting errors for each technique when using an up to 5mm fit. For an extended fit, the conic model does not require correction, however adding 0.25mm to the limbal radial position will vastly improve the accuracy of the spherical and biconic modelling techniques.

As the limbus and sclera are directly connected and share some geometrical properties, accurate scleral representation is vital for valid and reliable limbal modelling. The errors associated with the scleral fitting process indicate that despite their consistency, their magnitudes are relatively high when compared to the other fitting methods. As the sclera is the largest component of the eye, the size of this error will propagate through any of the other model components, including the limbus. This indicates that a possible method of mitigating the limbal fitting errors may be to modify the scleral fitting process. An alternative to the sphere fitting technique is to fit the sclera to a polynomial function. [74] Although this method will reduce the RMS fitting error around the limbus, it will never provide a realistic realisation of the entire ocular globe. This is due to the inherent nature of the function and the fact that the in-vivo topography can only be measured across a limited portion of the eye, thus making extrapolation beyond the measured region impossible. For this reason, when considering a whole eye model, the use of any fitting surface other than a sphere will be problematic. Although it could be viewed as a limitation, the sphere fitting technique is the most commonly used when producing whole-eye models. As this paper is presenting the current misrepresentation of the limbus, considering the error induced by this technique was key to highlighting the issues involved in modelling the ocular globe through current methods.

Additionally, the presented study did not consider the eye’s posterior surface and was limited to the anterior surface only. Unlike Scheimpflug or optical coherence tomography (OCT) based measuring devices, the ESP only takes measurements of the anterior surface of the cornea. This was not deemed to be a disadvantage as it is not uncommon for studies to exclude the posterior surface. [75–77] This is mainly due to the relatively small contribution of the posterior surface that provides only -5.9 D of the eye’s refractive power compared to 48.9 D from the anterior surface. [78] In addition to this, the measurement of the posterior surface is often deemed relatively unreliable. [28, 79–82]

The data presented in this paper suggests that the limbus is not being represented accurately through current modelling techniques. However, for the purpose of modelling, the most accurate method of representing the limbus is to use corneal topography data from the whole cornea in conjunction with the conic modelling technique. In doing so, the fitting errors can be minimised, and any residual errors can be accounted for by using correction factors based on the data provided. This will allow for a more accurate representation of the limbus, paramount for the fitting of mini-scleral and scleral contact lenses, and the customisation of the soft contact lenses. All types of lenses that can restore visual acuity in patients with irregular corneas, and address ocular pathologies such as keratoconus and ocular surface disease. [83, 84]

Additionally, it is evident that scleral geometry may be problematic when considering a whole eye model. Despite this, other modelling techniques are not currently feasible for generating realistic representations of the entire ocular globe. Advances in scleral modelling techniques will, therefore, be vital for the improvement of limbal representation. These developments will greatly enhance accuracy, which in turn will lead to improvements in the validity of ocular models.

## Supporting information

S1 Data Clinical data as measured by the ESP. (XLSX)

S2 Data Results dataset. (XLSX)

S3 Data Clinical limbus representation as measured by the ESP. (ZIP)

S4 Data Modelled limbus representation as generated by the conic model. (ZIP)
